# Synthesis and evaluation of bis(imino)anthracene derivatives as G-quadruplex ligands†

**DOI:** 10.1039/d0md00428f

**Published:** 2021-03-26

**Authors:** Tomris Coban, Cameron Robertson, Sianne Schwikkard, Richard Singer, Adam LeGresley

**Affiliations:** LSP&C, SEC Faculty, Kingston University Kingston-upon-Thames KT1 2EE UK a.legresley@kingston.ac.uk

## Abstract

The synthesis of a small number of bis(imino)anthracene derivatives is reported. They were evaluated *via* NMR for binding efficacy to the G-quadruplex-forming oligonucleotide sequence (TTGGGTT) and show activity against the HeLa cancer cell line. These novel ligands are compared to previously synthesised G-quadruplex ligands that target telomeres and oncogenes.

## Abstract 2

The synthesis of a small number of bis(imino)anthracene derivatives is reported. They were evaluated *via* NMR for binding efficacy to the G-quadruplex-forming oligonucleotide sequence (TTGGGTT) and show activity against the HeLa cancer cell line.

## Graphical abstract

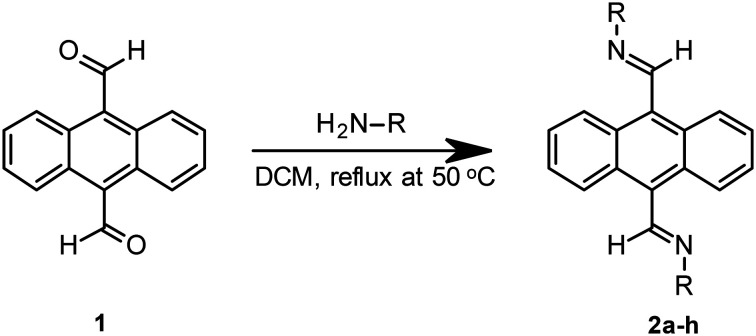

## Introduction

G-quadruplexes (G4) became biologically relevant in the 1980s mostly based on the seminal work undertaken by Dr. Elizabeth Blackburn which showed telomeric DNA forms non-Watson–Crick guanine–guanine base-paired intramolecular DNA structures.^1^ This discovery led to increased research interest and initiated interest in the field of G-quadruplex research.^2^ G-Quadruplex DNA structures heralded a new approach in anticancer drug design and development through the targeting of secondary DNA structures.^3^ It has been demonstrated that G-quadruplexes are present in genes that have been shown to be overexpressed in several different cancers, the stabilisation of which can effectively act as a switch for these genes.^4–8^ The first telomere interacting molecular structure was found to be 2,6-diamidoanthraquinone derivatives synthesised by Sun *et al.*^9^ These observations have led to an exponential growth in the development of synthetic small molecules for G-quadruplex binding and represent a rational approach to lead molecule design. Despite a large and diverse libraries of potential lead compounds having been reported, the search for G-tetrad selective compounds that can indirectly inhibit telomerase extension of DNA in cancer cells continues.

**Fig. 1:**
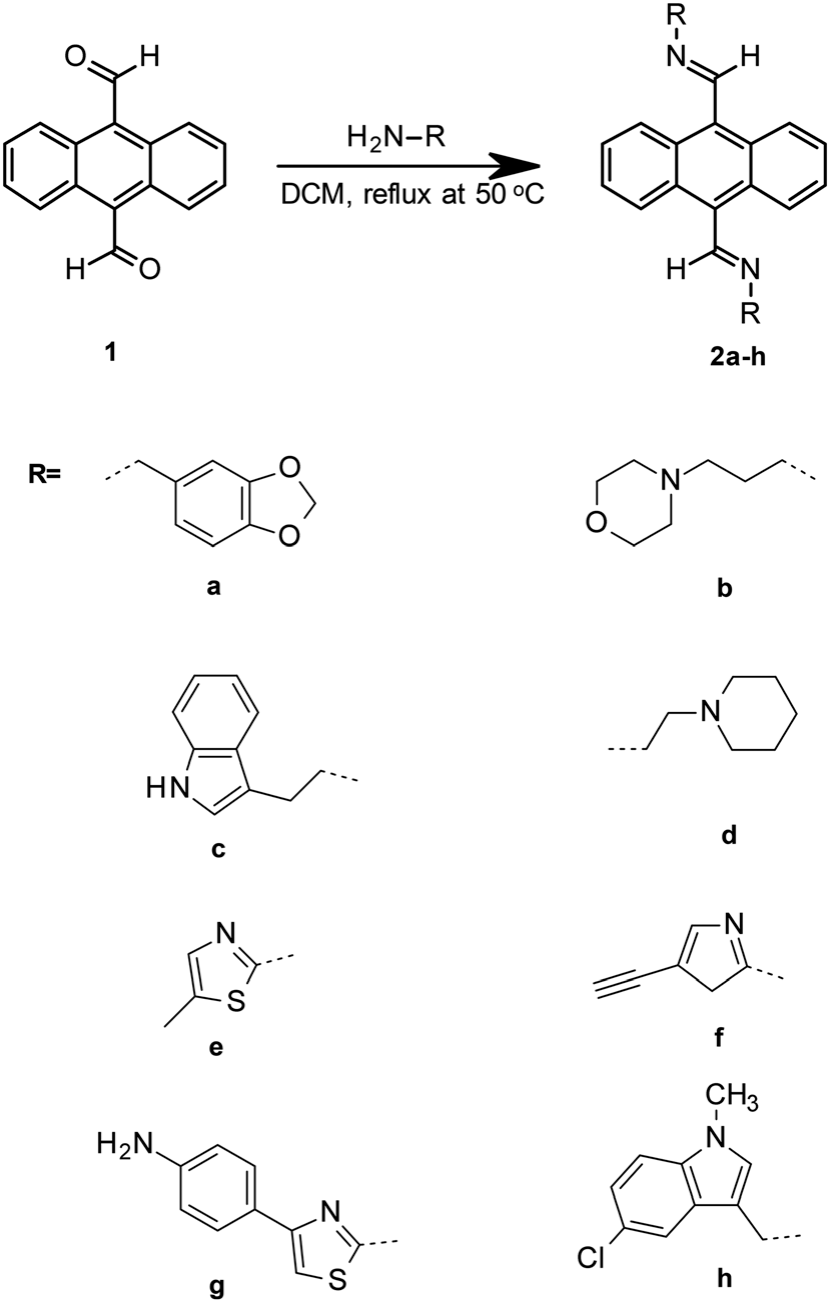
Simplified scheme for imine coupling to acrylate as done for novel imine coupled anthracene derivatives.

The principle motifs of a selective G-quadruplex ligand have been reported by a number of research groups.^10–14^ These include the requirement for planar aromatic systems and distal ionisable amine groups to interact with the phosphate backbone of the quadruplex DNA. Our intention was to couple a range of ionisable amine groups **a–h** to anthracene-9,10-dicarbaldehdye **1** and probe their selectivity for quadruplex over duplex DNA and to test them for cytotoxicity in HeLa cells (Fig. 1).

The reversible nature of the imine reaction facilitates the future establishment of a dynamic combinatorial library for this class of ligand, which may enable the *in situ* amplification of a specific (bis)imino compound from our anthracene derivative depending on the thermodynamics associated with exposure to quadruplex over duplex DNA. However, in this manuscript we report the formation and testing of a small static combinatorial library based on **1**.

## Results

Reaction of a series of amines to **1** was achieved using literature methods.^15^ The amines were selected owing to their potential for selectivity for the G-quadruplex *e.g.* Tryptamine is a known monoamino alkaloid which was selected based upon its amino group position in the ring system (see ESI† S1 and S2).

From Table 1, we show 3 compounds **2f**, **2g** and **2h** were not successfully synthesised due to solubility issues.

**Table 1:** Comparison of yields, experiment times and structures of specific imine coupled amine groups to anthracene core

Compound	Yield (%)	Time (h)
**2a**	78	24 h
**2b**	60	24 h
**2c**	80	12 h
**2d**	65	24 h
**2e**	75	24 h
**2f**	—	48 h
**2g**	—	48 h
**2h**	—	48 h

Before biochemical and *in vitro* analysis, the imines were tested for aqueous stability in Dulbecco's modified Eagle's medium (DMEM). Using an internal standard (TSP), after qNMR analysis at time point 0 and 24 h later no breakdown was observed with no significant concentration changes detected in triplicate experiments.^16^

**Fig. 2:**
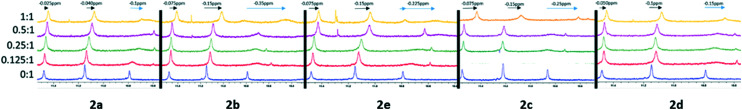
1D 1HNMR spectra, referenced to TSP, showing titration of drug compounds and *d*(TTAGGGT)_4_. A slow exchange interaction, is illustrated with black arrows and fast exchanging interactions by blue arrows preliminary experiments were done to screen for activity and synthesised compounds that showed activity were run in triplicate to get an average, SD and SEM values, from these results, it was shown that all 5 ligands showed cancer activity with IC_50_ values ranging from (∼18–31 μM) as illustrated in Fig. 3.

Compounds **2a–2e** were evaluated for G-quadruplex interaction using NMR titration studies with *d*(TTGGGTT)_4_ with intercalation comparisons made against doxorubicin and nemorubicin (see ESI† S1). Previous literature with *d*(TTGGGTT)_4_ has demonstrated that G-quadruplex ligands intercalate specifically at the point between G5–T6.^17^ Large broadening of G5 imino proton peaks, accompanied with an up-field shift, shows this. We also see the peaks for T6 (methyl, aromatic and ribose) shifting to a lower field in accord with the up-field shift of the G5 peaks. This change is explained by the formation of a complex between the ligand and the tetrad formed at G5, which pushes the T6 unit away from the guanine π-system. This intercalation framework agreed with ESI/MS carried out which illustrated a 1 : 1 complex.^17^ We see in Fig. 2, much like previous work, that line broadening and up-field shift occurs at peaks representative of proton G6NH (10.79 ppm) significantly, alongside the significant peak broadening and down field shifting of T6H6/8 and T6CH3 peaks with the addition of proposed ligands.^18^

**Fig. 3:**
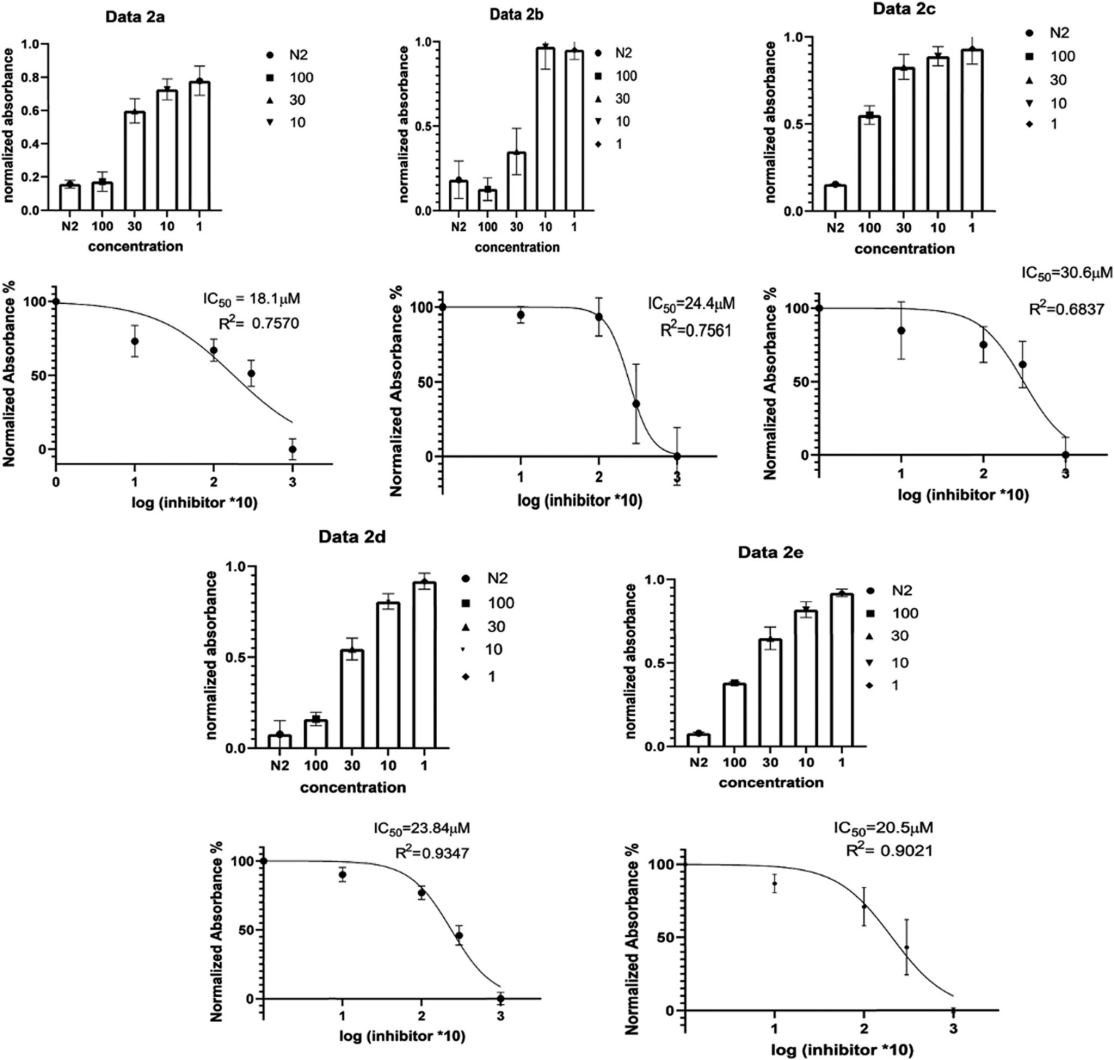
Cell culture, presto blue assay results for **2a**, **2b**, **2c**, **2d**, **2e**. The data shown is after 24 hour incubation of drug treated HeLa cells. Top chart illustrates mean and SEM of HeLa cell viability at concentrations 100 to 1 μM with positive control (doxorubicin (N2)). In the top charts, values are normalized to negative control (DMSO) at a value of 1 (cell viability). The bottom charts then show the normalized absorbance percentage to the negative control (percentage cell viability) of log inhibitor concentration (multiplied by 10 to account for 1 μM concentration) IC_50_ values and *R* squared values are included in the chart. Error bars indicates standard error of mean from *n* = 3 experiments.

These significant changes demonstrate distinctly different bound and unbound forms through large broadening effects at a ratio of 0.5 : 1. There is also subtle line broadening and up-field shifting of G4NH (11.48 ppm) and G5NH (11.15 ppm) for each ligand that shows chemical exchange between two similar environments of bound and unbound ligand. Maximum line broadening occurs at a ratio of 1 : 1 drug to G-quadruplex and chemical shift difference changing up to 1 : 1 with the bound and unbound peaks being present. This matches the effect seen by nemorubicin intercalation with *d*(TTGGGTT)_4_ in previous literature suggesting a 1 : 1 stoichiometry. This investigation showed that the five imine coupled ligands showed G-quadruplex intercalation with 1 : 1 stoichiometry matching that of the current cancer drugs doxorubicin and nemorubicin (Table 2).

**Table 2:** Chemical shift differences for G-quadruplex peaks indicative of ligand interaction

Compound	Difference from 1.58 ppm (ppm) T6CH3	Difference from 7.23 ppm (ppm) T6H6/8	Difference from 11.55 ppm (ppm) G3NH	Difference from 11.22 ppm (ppm) G4NH	Difference from 10.86 ppm (ppm) G5NH
**2a**	0.09	0.17	−0.04	−0.08	−0.2
**2b**	0.093	0.22	−0.066	−0.135	−0.34
**2e**	0.146	0.19	−0.056	−0.11	−0.24
**2c**	0.157	0.126	−0.06	−0.115	−0.25
**2d**	0.094	0.16	−0.03	−0.07	−0.13

Compounds **2a–2e** were subsequently tested against a HeLa cancer cell line (HeLa, American Type Culture Collection (ATCC) (ATCC® CCL-2™)) and they were all prepared in 100, 30, 10 and 1 μM concentrations so that IC_50_ values could be calculated to establish whether drugs were cytotoxic to HeLa cells (see ESI† S1).

## Discussion

The ligands have different functional groups present in their side chains; 1,3-benzodioxole, propylmorpholine, 2,5-dimetylthiazole, 3-methyl indole and piperidine respectively, which we suspected stabilise the hydrogen bond between the tertiary amine and oxygens of phosphate. However, all these groups have been shown in previous studies to demonstrate DNA and G-quadruplex intercalation alongside anti-cancer activity. This suggests that each of these groups may have more of an effect on IC_50_, than solely the effects on hydrogen bond stabilisation.

We see that the two groups can be grouped by their absence of oxygen or sulphur, piperidine and 3-methyl indole show quite divergent differences in ppm between bound and unbound forms of the G4 model. These two compounds also have 2 of the higher IC_50_ values of the 5 intercalating compounds, 24 μM (**2d**) and 30.5 μM (**2c**).

Previous studies have demonstrated non-selective binding to DNA and G4 structures where the mode of binding to DNA, like our results, showed a 1 : 1 stoichiometry, with Gibbs free energy calculations showing a spontaneous intercalation which suggests a stabilising, energetically favourable reaction.^19^ It was also shown that using piperidine as a *N*-propylamino attached to carbazoles gave the highest stabilizing ligands in a study.^20^ Interestingly, however they did identify that there was an inverse correlation between G-tetrad stabilization and G4 DNA selectivity.^21^ This is very interesting for our results, as it could begin to explain why we see that the relationship between the change in ppm does not correlate completely with IC_50_ values, even showing an inverse relationship to what was expected from nemorubicin results.^17^ It could show that G4 ligands intercalate and stabilise very effectively but IC_50_ will be increased as G4 ligand is less selective for G4 structures. This could explain why we see piperidine having similar chemical difference effects and stabilization effects to other compounds but has a higher IC_50_. 3-Methyl indole is less investigated for this purpose because of its role primarily as an odourant. There are however genetic studies that show protective mechanisms against damage by 3-methyl indole derivatives that can induce apoptosis at low concentrations. This was shown in a study by Nichols WK they looked at 3-methyl indole mediated cytotoxicity in human epithelial lung cells, with CYP2F1 over expressed.^22^ This is relevant to this study as it shows that 3-methyl indole has a structure activity relationship which relates to cytotoxicity of genetically impaired cells, which could possibly explain anti-cancer activity.^22^ The anti-cancer activity of indoles has also been shown with indole groups in isoxazolo[5′,4′:5,6]pyrido[2,3-*b*]indoles, which showed significant anticancer activity comparable to standard drugs however the mechanism of action and structure activity relationship is yet to be investigated.^23^

**Fig. 4:**
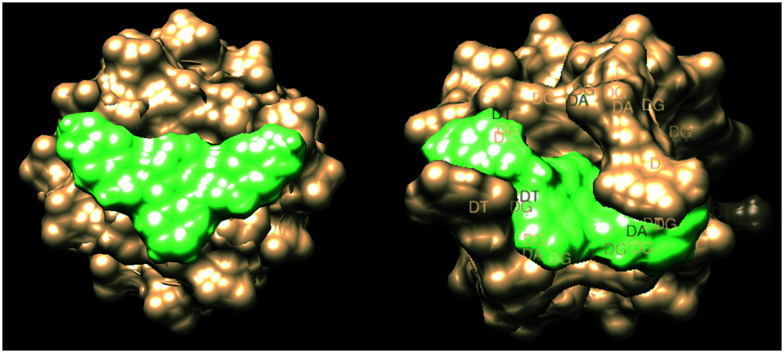
Surface energy model of ligand **2a** intercalating parallel (PDB entry139d) and antiparallel (PDB entry 6IMS) G-quadruplex strands (UCSF Chimera 1.15).

The three groups that contained oxygen, sulphur and nitrogen, and nitrogen and oxygen, 1,3-benzodioxole, 2,5-dimethylthiazole and *N*-propylmorpholine respectively, showed a close linear correlation between their ability to stabilise G4 structure and IC_50_. They also, as a group had the best IC_50_ values with 1,3-benzodioxole and 2,5-dimethyl thiazole as the best candidates for further testing. Further synthesis and reinforcement of DNA binding potential of 1,3-benzodioxole has been illustrated by a study which showed DNA binding was moderate to mild and suggested that cytotoxic effects were through a different mode of action, which supports the selectivity argument for 1,3-benzodioxole.^24^ They demonstrated through molecular docking that a benzodioxole derivative had potent telomerase inhibition alongside potent anticancer activity in human gastric and human melanoma cell lines.^25^ This shows that this group may add additional benefit through telomerase inhibition in a different method of action to the one investigated here with G4 stabilisation. The 2,5-dimethylthiazole has been studied extensively in the form of the fluorescent marker thiazole orange for its intercalation potential. It was shown in early research that thiazole orange binds both as a monomer and dimer to DNA. Monomers of thiazole orange stack between DNA bases. It is also shown as the main way of binding with duplex DNA. Interestingly they also reported binding to poly(dG) which showed binding as a monomer and dimer as well. This supports our results showing intercalation and stabilisation of G4 as seen in our results.^26^ Selective stabilization of G4 structures by thiazole has further been supported more recently with studies that showed 1 : 1 stoichiometry between dye and G-tetrads in quadruplex DNA. It also illustrated that there was a tight complex formed between the two. Interestingly they showed that in the presence of K^+^ ions, this binding seemed to disappear.^27^ To attempt to rationalise the chemical shift difference and IC_50_ values observed, computational modelling of the 5 ligands with parallel (PDB entry139d) and antiparallel (PDB entry 6IMS) quadruplex structures was undertaken using Autodock Vina both in conjunction with UCSF Chimera 1.15v and CB-Dock – these values are show in Table 3.^28–30^ The docking box was chosen within Chimera to encompass the entire quadruplex model, thus allowing Vina to search the whole of the target space. The docking box was larger than the upper size recommended for use with Autodock Vina. Therefore blind docking was also carried out using the CB-Dock web server, which uses cavity detection based on analysis of the solvent accessible surface to identify potential docking sites in the target, followed by the use Autodock Vina to generate and score the ligand poses at the detected dicking sites. Because the same scoring function is used by both methods the docking scores are directly comparable. The results are very close to those from Autodock Vina and Chimera which forgoes the cavity detection step. Details of the parameters and programs used can be found in ESI† S1. Energy minima scores were obtained and are shown against IC_50_ and Δ*δ* values shown in Table 3. Interestingly, ligand **2a** gave the best apparent fit to both quadruplex structures as indicated in Fig. 4. It should be noted that there is a correlation between IC_50_ and all of the docking data, with the exception of **2c**, which appears to be an outlier. There appears to be preferential binding to the end of the quadruplex as opposed to intercalation. There is further evidence of groove binding.

**Table 3:** Comparison of IC_50_ values and G quadruplex chemical shift differences for compounds **2a–2e** alongside Autodock Vina blind docking scores (kCal mol^−1^) using Chimera and CB-Dock for parallel and antiparallel quadruplex models. Shown in order of decreasing IC_50_

Compound	Parallel docking score		Antiparallel docking score			
	Chimera	CB-Dock	Chimera	CB-Dock	Δ*δ* (ppm)	IC_50_ (μM)
**2c**	−6.8	−7.2	−7.3	−7.8	0.25	30.6
**2b**	−6.4	−6.3	−6.2	−6.5	0.34	24.4
**2d**	−6.6	−6.9	−6.4	−6.6	0.13	23.84
**2e**	−6.7	−6.8	−6.4	−7.0	0.24	20.5
**2a**	−8.1	−8.1	−7.7	−7.8	0.2	18
Doxorubicin^17^					0.31	0.374
Nemorubicin^17^					0.31	0.08
RHPS4 (ref. 32)					—	0.2
BRACO19 (ref. 33)					—	5.25
Quarfloxin^34^					—	4.44
BMH-21 (ref. 34)					—	0.46
CX-5461 (ref. 34)					—	6.89

Compound **2b**, which has a propylmorpholine group has been implemented in the design of compounds for telomeric and oncogenic G-quadruplex affinity with varying degrees of success. It was shown through molecular docking analysis that compounds made with propylmorpholine interact with the groove and or loop of the G-quadruplex, which supports our stacking hypothesis through interaction with these external components. This study also showed inhibition in the growth of A549 cancer cell lines with low cytotoxicity to normal cells. This study concluded that addition of propylmorpholine to naphthalimide supports tumor cell cytotoxicity selectivity.^21,31^

Looking at Table 3, we see the IC_50_'s of doxorubicin and nemorubicin are much lower than the IC_50_ values of our synthesised compounds, however we do see that their Δ*δ* is comparable to our two lowest IC_50_ compounds. This suggests a similar interaction mechanism where a 1 : 1 stoichiometry, with planar stacking on the G6 tetrad.^17^ This however does show that G-quadruplex binding may start through this stacking and be an initiating step for cytotoxicity, but we see from these results that side chains give comparable chemical shift differences and intercalation stoichiometry but produce very different IC_50_ values. This however could be explained by selectivity measures. Comparisons of other G-quadruplex ligands and their IC_50_ values to synthesised ligands shows promise for these compounds as scaffolds for drug discovery as we see the synthesised compounds IC_50_ values are within the same order as previously synthesised G-quadruplex ligands.

## Conclusions

The promotion of quadruplex DNA formation as observed in solution by NMR, and initial biological testing, suggests the class of G-quadruplex ligand based on imine coupled ligands possess an anticancer activity. Whilst, compared to other synthesised G-quadruplex ligands, we see a poorer IC_50_ value for these preliminary imine coupled anthracene derivatives, the propensity for intercalation to G-quadruplex and simple synthesis allows for extensive expansion of imine coupled anthracene derivatives as scaffolds for further modification and drug discovery. Whilst this communication expands incrementally on the planar anthracene arrays, already evaluated for G-quadruplex stabilisation, there is a requirement for further investigation to identify the specific G-quadruplexes targeted. *E.g.* telomere or oncogene promoter regions. However, as discussed in a recent review much of the efforts over the past decade have been focused on achieving selectivity for a specific G-quadruplex.^35^ Despite this, there is evidence that the most clinically efficacious G-quadruplex ligands are those that target multiple types of G-quadruplexes, such as in the case of triarylpyridine 20A.^36^

Whilst there are a number of G-quadruplex ligands currently in clinical trials, there are, as yet no ligands approved for clinical use. The formation of more G-quadruplex structures during the S phase of the cell cycle in neoplasia continues to endorse the need for a greater range of potential ligands, both to probe selectivity and as lead compounds for therapeutic agents as more is understood about how these structures are modulated in human cells at different points within the cycle, having a diverse range of potential ligands may help to make personalised cancer chemotherapy a reality.

## General preparation example – **2c**

Tryptamine (390 mg, 0.00245 mol) and 9,10-dibromocarbaldehyde (250 mg) were stirred in dry DCM (40 ml) over molecular sieves. The reaction mixture was refluxed for 12 h under N2 environment. The resultant mixture was monitored *via* TLC which showed the consumption of both starting materials. After TLC analysis, the crude mixture was left to cool down to room temperature. The resulting yellow solid **2c** was filtered and it was dried under vacuum before being recrystallised from methanol to give (0.44 g, 80%) yield. 1H NMR (400 MHz; d6-DMSO) *δ* = 3.68 (4H, t, *J* = 6.65 Hz, CH-8) 4.26 (4H, t, *J* = 8.66 Hz, CH2-7), 7.01 (2H, t, *J* = 8.76 Hz, CH-6), 7.10 (2H, t, *J* = 8.78 Hz, CH-5) 7.23 (2H, s, CH-4), 7.39 (2H, dddd, *J* = 8.0, 1.2, 0.5, 0.5 Hz, H-10), 7.424 (4H, dd, *J* = 6.63, 2.21 Hz, CH-3), 7.695 (2H, d, *J* = 7.74 Hz, CH-9), 8.123 (4H, dd, *J* = 6.97,2.68, CH-2)13C-NMR (600 MHz; d6-DMSO) *δ* = 22.5, 46.9, 111.6, 112, 118.7, 120.2, 121.7, 122.2, 126.7, 127.5, 127.6, 130.7, 130.8, 136.4, 159.9 IR (ATR): *v* = 2843.46, 2922.6, 1652.99, 1621.80, 741.77 755.12 HR MS (ES) *m*/*z* = found 519.2541, requires 520.6642 [M + 2H]+.

## Conflicts of interest

There is no conflict of interest to declare.

## Supplementary Material

MD-012-D0MD00428F-s001
