# Supplementary material for: Synthesis and evaluation of bis(imino)anthracene derivatives as G-quadruplex ligands
Source: RSC Med Chem. 2021 Mar 26;12(5):751–7. doi: 10.1039/d0md00428f (PMC8152782; doi:10.1039/d0md00428f)
Supplement: MD-012-D0MD00428F-s001 [file MD-012-D0MD00428F-s001.pdf]

Electronic Supplementary Material (ESI) for RSC Medicinal Chemistry.
This journal is © The Royal Society of Chemistry 2021

Supplementary Information

S1. Materials and methods

All chemicals were purchased from Sigma-Aldrich or Fisher scientific. Anhydrous reactions were performed using dry glassware which was flame dried under vacuum and carried out under an inert nitrogen environment. Amine groups a-h were obtained from Sigma-Aldrich and used without further purification. All other chemicals were purchased from the Aldrich Chemical Company.

All NMR spectra were acquired with a Brüker AV400 FT-NMR spectrometer and a Bruker AV600 FT-NMR Spectrometer. All NMR spectra were acquired with high throughput 5mm NMR tubes o/d, No Z107374, Brüker BioSpin AG, Switzerland. All NMR peak chemical shifts are in ppm relative to an internal standard Trimethylsilylpropanoic acid (TSP) at $\delta = 0.00$. All coupling constants use the units of Hertz (Hz). Spectral acquisitions were sweep width optimised in 1D 1H spectra and optimised parameter sets specific to 2D NMR (HSQC and COSY) were used. The data was automatically processed with the window function, linear prediction, zero-filling, phasing and baseline optimisation set within TopSpin 3.5 pl7 (c) 2017 Brüker BioSpin.

Infra-red analysis was performed using the neat oil or solid powdered sample coated onto ATR disc which is analysed with a ThermoScientific Nicolet iS5 FT-IR spectrophotometer. Low resolution mass spectra (LR MS) and High-resolution mass spectra (HR MS) were acquired by MEDAC Ltd. This was done in accordance with BS EN ISO 9001:2008 provision for microanalysis service.

NMR intercalation procedure

NMR tubes used were Wilmad 5 mm, thin-walled tubes obtained from GPE Scientific (Product No: 502-7). NMR samples were spiked with Sodium d4-trimethylsilylpropionate (TSP) to a concentration of 10 mmol/L. All spectra were recorded in 0.6ml of H_2O-D_2O (90:10 v/v) solvent, with 25mM phosphate buffer, 150mM KCl and 1mM EDTA.

Oligonucleotide d(TTAGGGT)4 was purchased from Sigma Aldrich and made up to a concentration of 0.4mM in the solvent mix. This oligonucleotide solvent mix was then micro-pipetted at a volume of 0.6ml into NMR tubes. Stock solutions of drug compounds in DMSO-d_6 were used to make up the molar ratios of 0.125:1, 0.25:1, 0.5:1 and 1:1 drug to oligomer. A non-active drug (TC102) and DMSO on its own were added as controls to validate that the effects on the spectra were from cytotoxic elements of the drug compounds rather than the effects from DMSO or addition of compound, which could change environment, pH or shimming of NMR instrument.

Spectra were acquired at 300 K using a Bruker Avance III 600MHz FT-NMR. The WATERNOESY parameter set which executes the pulse sequence noesygppr1d (Bruker, size of fid 65536, 12335.526 Hz spectral width, 128 scans, d1 = 4 s) was used to acquire ^{1}H 1D experiments with a pre-saturation delay during relaxation and mixing time (experiment time: 14 min 44 sec). With this pulse sequence, all non-exchangeable protons are detected in the deuterated D_2O/H_2O buffer solution. T1 relaxation experiments were acquired for d1 calibration with a modified version of the zggpw5 pulse sequence. With a saturation recovery block (delays 0.01, 0.22, 0.54, 1.05, 1.84, 3.08, 5.01, 8.01, 12.69, 20.00 s) before the first 90° pulse and using water suppression (Bruker, size of fid 65536/11 F2/F1, 12335.526/5882.353 Hz spectral width in F2/F1, 64 scans, 0.1 s relaxation delay) (experimental time 1 h 4 min 6 sec).

Bruker Topspin 3.5 pl7 was used for assignment of peaks, manual phasing, baseline correction and manual integration. Manual integration was done to ensure bias and slope was correct and that the integrals were all of the same width. Calculation of chemical shift change done through built in top spin distance measurements between peaks.

Presto Blue Assay

Cell suspension (200µl) containing 5000 healthy HeLa cells was seeded in 96-well plates (BDFalcon, Franklin Lakes, NJ) and allowed to attach and stabilize overnight at 37 °C with 5% CO_2. Aspiration of media and rinsing solution was done through pipetting off liquid. Cell viability was then tested with PrestoBlue assay.The presto blue assay was carried out in light sensitive conditions. 100µl of PrestoBlue solution (10% in medium) was added to each well and the plates were incubated at 37 °C for 1 hour. After incubation time, the change in florescence was measured at 570/600 nm using a Spectra Max fluorescence multi-well plate reader.

Dissociation constants were calculated using the curve-fitting software Graphpad Prism 8.

Cell Culture Methods

Incubation was done using Thermofisher Steri-Cycle CO_2 Incubators and for plate reading Thermofisher Epoch Microplate Spectrophotometer was used. Centrifugation was done by Thermo Scientific Sorvall ST 40 Centrifuge Series.

Media Preparation

Commercially available DMEM (Dulbecco's Modified Eagle Medium- low glucose), including sodium pyruvate and α-glutamine, 500 mL, was obtained from Gibco, USA. All procedures carried out under sterile conditions, 94 mL of media was taken and the required volume of supplements (10 % Fetal Bovine Serum (FBS), 2 mM/mL Glutamine, 100 U/mL penicillin and 100 µg/mL streptomycin.) were added.

Cell Line and Cell Culture Conditions

Human cervical cancer cell lines (HeLa) were obtained from the American Type Culture Collection (ATCC) (ATCC® CCL-2™). HeLa cells were cultured in Dulbecco's Eagle Modified Medium low glucose (DMEM) containing 10 % Fetal Bovine Serum (FBS), 2 mM/mL Glutamine, 100 U/mL penicillin and 100 µg/mL streptomycin.

These cells were kept in media which was changed with fresh media every 2 days. All of the cells were cultured in a humidified incubator whose conditions are 37 ^{o} C, 5 % CO_2, 95 % air.

Cell Passaging

The cells were split when the cells reached 70 % confluency in the flask. First of all, the medium was removed from the flask and then cells were washed with DPBS (1X) (pH 7.4) containing 10 mM sodium dibasic phosphate, 2.7 mM KCl, 2 mM Potassium phosphate, 137 mM NaCl. After that, the cells were detached from the flask with 0.25 % (w/v) trypsin- 5mM EDTA in PBS (Phosphate Buffered Saline) solution and incubated for 5 minutes in an incubator with conditions at 37 ^{o}C, 5 % CO_2, 95 % air. Dissociated cells were resuspended with medium containing 10 % Fetal Bovine Serum (FBS) to inhibit the trypsin activity and then centrifuged at 1500rpm for 5 minutes. After centrifugation, the supernatant was discarded, and cells were resuspended with media and seeded into the flasks.

Cell Counting and Viability

In order to determine cell density a hemacytometer was used, with the following procedure. First of all, chamber and cover slip cleaned with 70 % IMS. After gentle drying, the coverslip was placed in position. 10 µL of the harvested cells in media (cell suspension) and a 10µl of a prepared 0.4% trypan blue in buffered isotonic salt solution (pH 7.2 – 7.3) were added to eppendorfs. 10µL of Eppendorf solution is then loaded into the hemacytometer. The chamber placed in the inverted microscope with 10x objective. Finally, the blue stained cells and total number of cells in the central gridded square were counted and the average number of cells per mL was estimated by applying this formula;

$$Concentration = \frac{Number\ of\ cells}{Number\ of\ squares}\ x\ 10000\ x\ sample\ dilution$$

With cell viability calculated with the following equation, where 95% viability was the target for cells;

$$\%viable\ cells = \left(1 - \left(\frac{Number\ of\ blue\ cells}{Number\ of\ total\ cells}\right)\right) x\ 100$$

Cell Freezing

Cells were trypsinized from the cell plate and then centrifuged as described previously in Section 3. After that, the supernatant was discarded and the pellet was resuspended with freezing medium, including 90% heat inactivated Fetal Bovine Serum (FBS) and 10 % Dimethyl Sulfoxide (DMSO). 1 ml of cell suspension were put into each cryovial and then frozen at -80 ^{o}C or kept in liquid nitrogen tank for long term preservation.

Cell Thawing

Cryovials were taken from the liquid nitrogen tank and thawed quickly at 37 ^{o}C. After that, the cell suspension was transferred to a 15 mL tube and 5 mL of the medium was added into the cell suspension drop by drop with gentle shaking. Centrifugation was done at 1500rpm for 5 min and then supernatant was discarded. After the pellet was resuspended with medium, they were seeded to T-25 flasks. Subsequent to 12 hours incubation, the media was removed and washed with DPBS (1X) (pH:7.4) containing 10 mM sodium dibasic phosphate, 2.7 mM KCl, 2 mM Potassium phosphate,137 mM NaCl. Fresh media was added to the cell monolayer a day later thawing.

Seeding plates

Assays were carried out using a 96 well plate. This 96-well plate contains one blank, 3 controls which contains cells including a negative control with media, one vehicle control which is DMSO and medium solutions, and a positive control of Doxorubicin (200µL) and media. Drugs were prepared in 100, 30, 10, 1 µM concentrations to make 8 seeded well plates in total which were done in triplicate. All drugs were prepared in DMSO and then diluted in media to give 5µL of DMSO max in 100µM treatments which is controlled by vehicle with a DMSO volume of 5µL.

Computational modelling

Ligands were drawn using Chemdraw 19.1 PerkinElmer Informatics Inc, with the 3D translation carried out using Avogadro 1.20, which was further used to remove water, add hydrogens dependent on pH 6.7. Force field geometry optimisation was applied to structures before writing as pdb files for UCSF chimera v1.15 software (University of California), or using semi empirical molecular orbital theory (PM6) carried out using the Scigress (Fujitsu) computational chemistry package. Ligand docking was carried out using Autodock Vina using the Chimera interface with the parameters below, and using the CB-Dock web server. Autodock Vina analysis was undertaken with the below settings applied for the writing of pdbqt files.

All receptor options were set to true except for “Ignore chains of non-standard residues and Ignore all non-standard residues” set to false. Ligand options were set to false. Binding modes = 10, Exhaustiveness of search = 8 and Maximum energy difference was set to 3kcal/mol. The docking box was chosen to encompass the entire quadruplex model, thus allowing Vina to search the whole of the target space. The docking box was larger than the upper size recommended for use with Autodock Vina, so blind docking was also carried out using the CB-Dock web server, which detects potential docking sites in the target by analysis of the solvent accessible surface and subsequently uses Autodock Vina to generate and score the ligand poses at the detected dicking sites. The results were comparable to those found using Autodock Vina and Chimera which forgoes the cavity detection step.

Docking data

Parallel G-quadruplex d(TGGGGTT)4 – PDB accession number 139D

2a

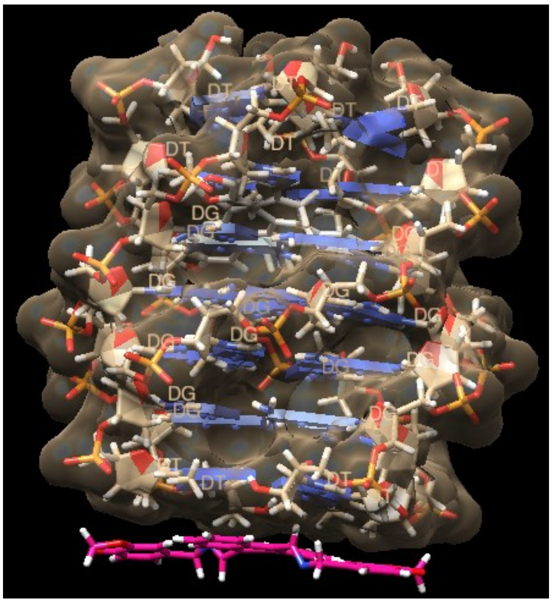

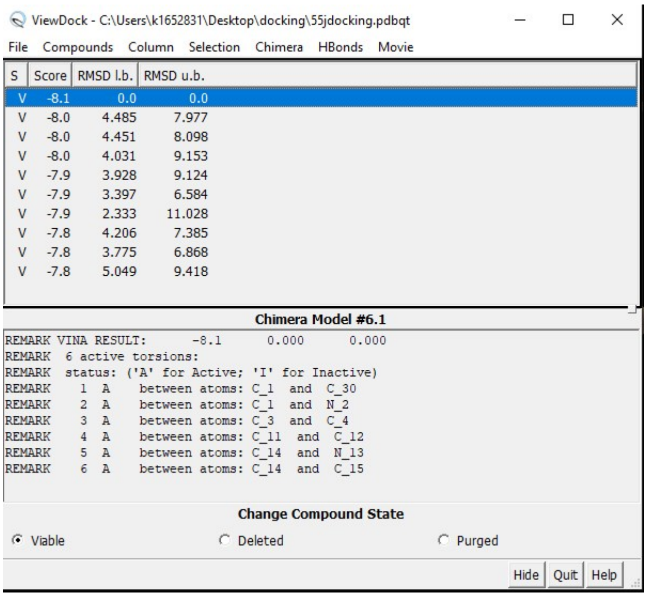

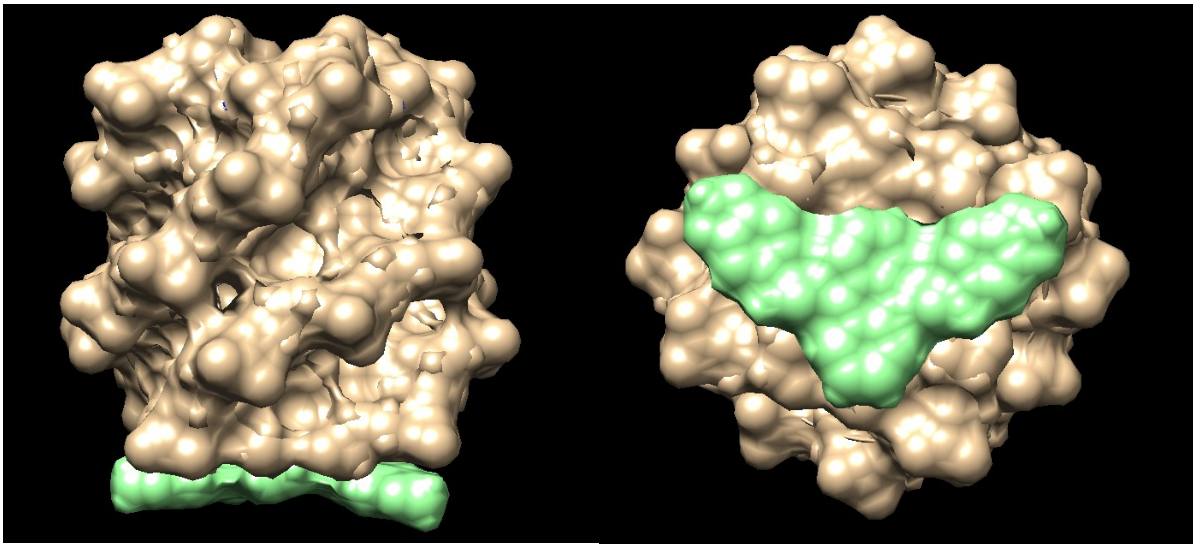

2b

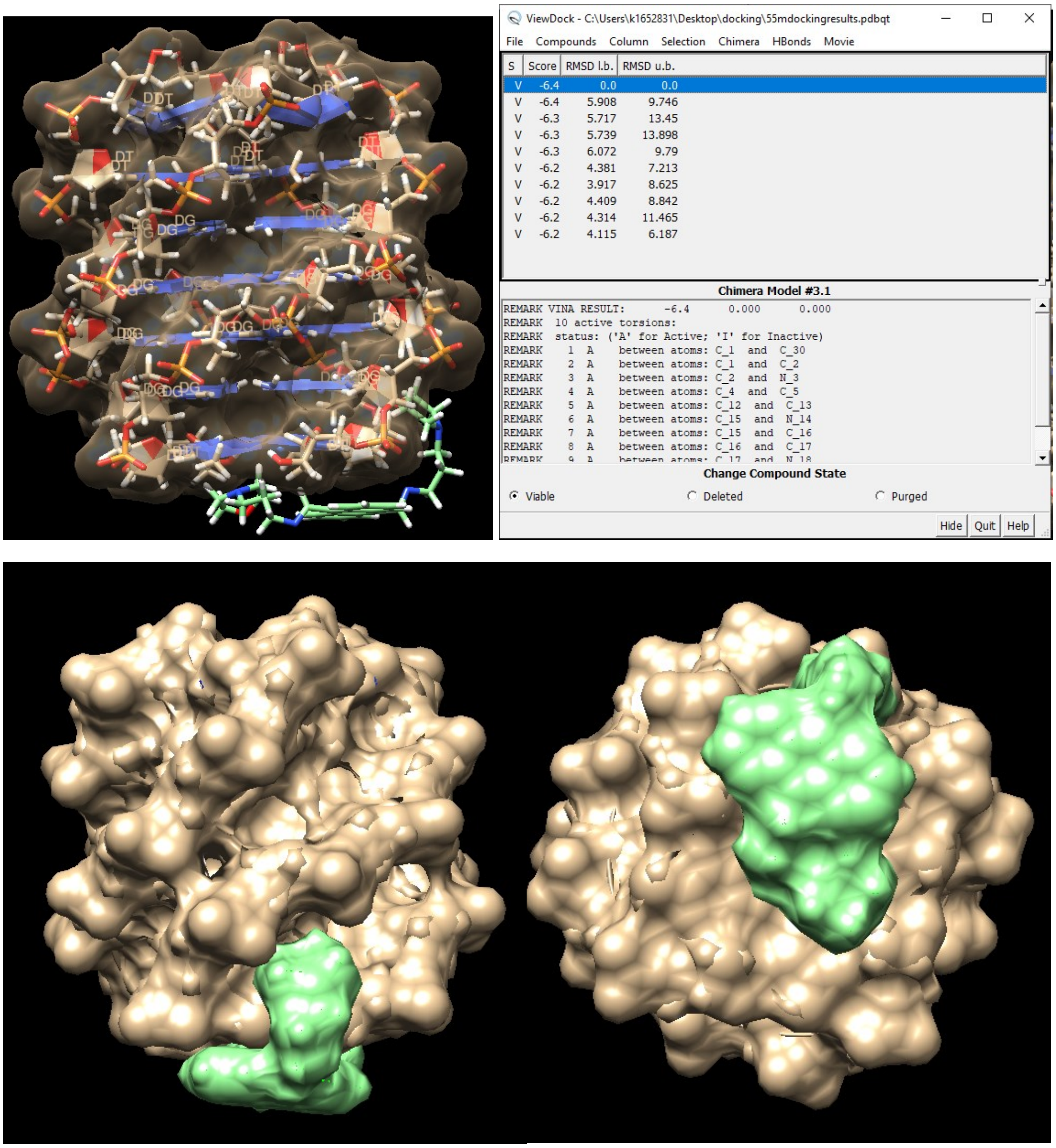

ViewDock - C:\Users\k1652831\Desktop\docking\55mdockingresults.pdbqt
File Compounds Column Selection Chimera HBonds Movie
S Score RMSD l.b. RMSD u.b.
V -6.4 0.0 0.0
V -6.4 5.908 9.746
V -6.3 5.717 13.45
V -6.3 5.739 13.898
V -6.3 6.072 9.79
V -6.2 4.381 7.213
V -6.2 3.917 8.625
V -6.2 4.409 8.842
V -6.2 4.314 11.465
V -6.2 4.115 6.187
Chimera Model #3.1
REMARK VINA RESULT: -6.4 0.000 0.000
REMARK 10 active torsions:
REMARK status: ('A' for Active; 'I' for Inactive)
REMARK 1 A between atoms: C_1 and C_30
REMARK 2 A between atoms: C_1 and C_2
REMARK 3 A between atoms: C_2 and N_3
REMARK 4 A between atoms: C_4 and C_5
REMARK 5 A between atoms: C_12 and C_13
REMARK 6 A between atoms: C_15 and N_14
REMARK 7 A between atoms: C_15 and C_16
REMARK 8 A between atoms: C_16 and C_17
Change Compound State
Viable
Deleted
Purged
Hide
Quit
Help

2c

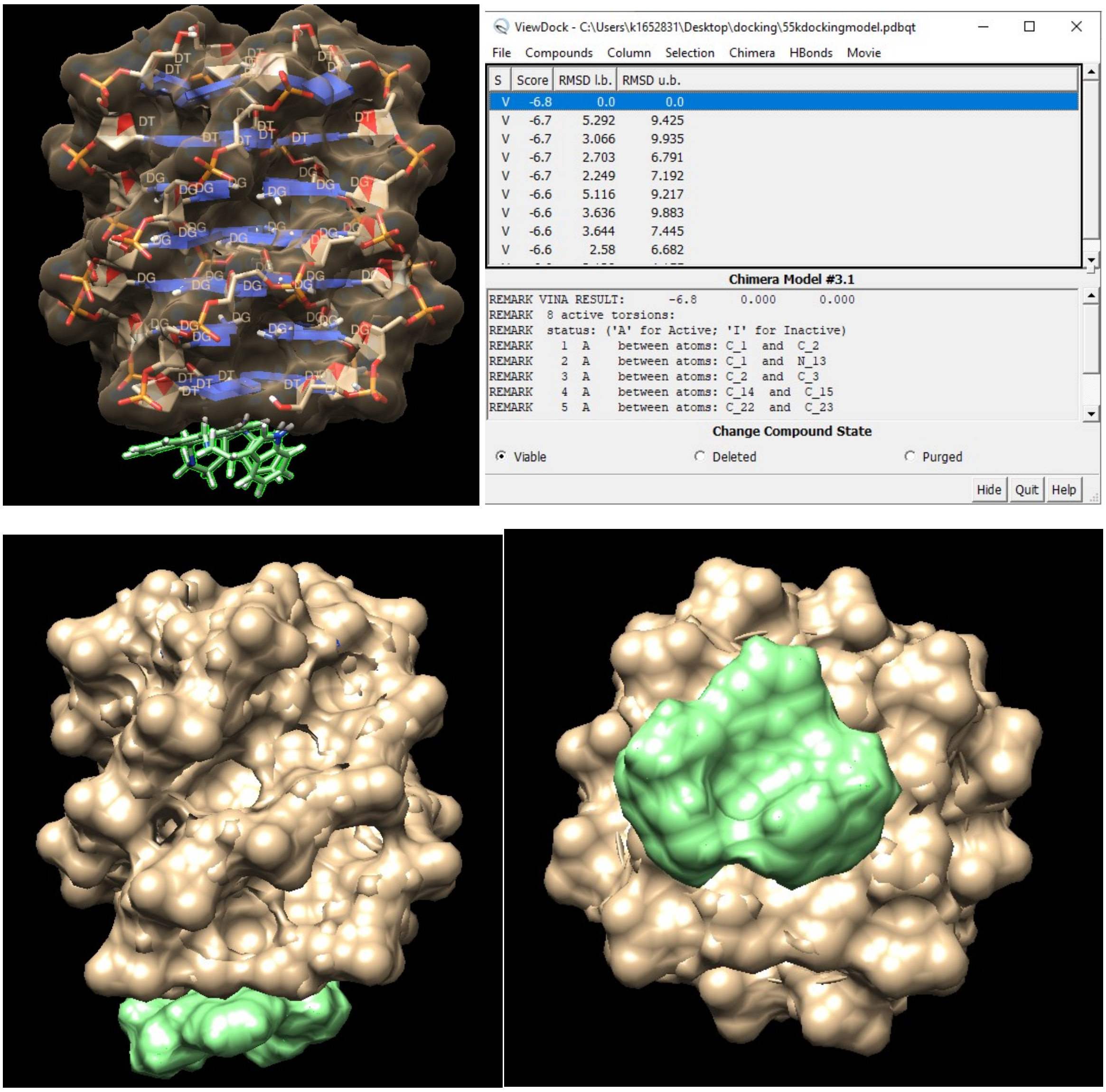
ViewDock - C:\Users\k1652831\Desktop\docking\55kdockingmodel.pdbqt
File Compounds Column Selection Chimera HBonds Movie
S Score RMSD l.b. RMSD u.b.
V -6.8 0.0 0.0
V -6.7 5.292 9.425
V -6.7 3.066 9.935
V -6.7 2.703 6.791
V -6.7 2.249 7.192
V -6.6 5.116 9.217
V -6.6 3.636 9.883
V -6.6 3.644 7.445
V -6.6 2.58 6.682
Chimera Model #3.1
REMARK VINA RESULT: -6.8 0.000 0.000
REMARK 8 active torsions:
REMARK status: ('A' for Active; 'I' for Inactive)
REMARK 1 A between atoms: C_1 and C_2
REMARK 2 A between atoms: C_1 and N_13
REMARK 3 A between atoms: C_2 and C_3
REMARK 4 A between atoms: C_14 and C_15
REMARK 5 A between atoms: C_22 and C_23
Change Compound State
Viable
Deleted
Purged
Hide
Quit
Help

2d

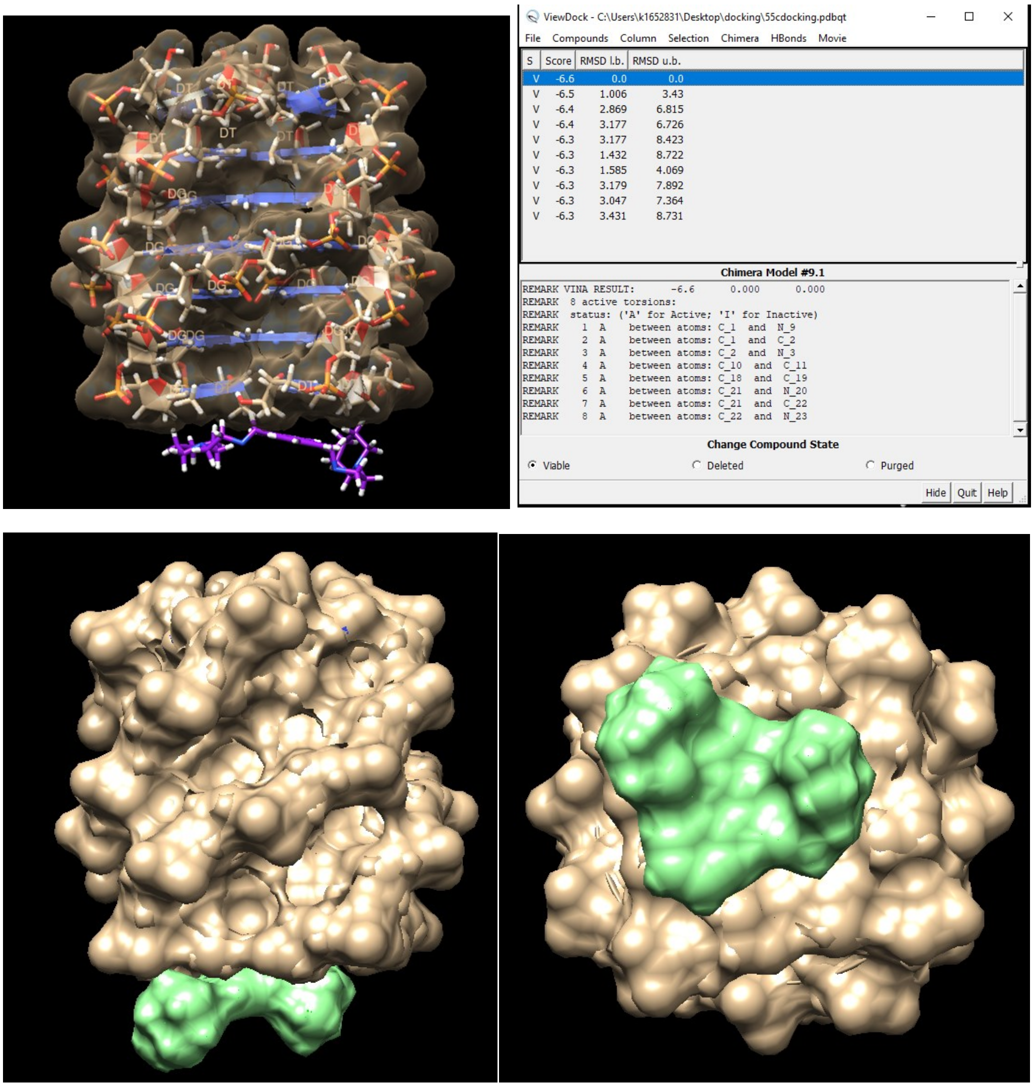
ViewDock - C:\Users\k1652831\Desktop\docking\55cdocking.pdbqt
File Compounds Column Selection Chimera HBonds Movie
S Score RMSD l.b. RMSD u.b.
V -6.6 0.0 0.0
V -6.5 1.006 3.43
V -6.4 2.869 6.815
V -6.4 3.177 6.726
V -6.3 3.177 8.423
V -6.3 1.432 8.722
V -6.3 1.585 4.069
V -6.3 3.179 7.892
V -6.3 3.047 7.364
V -6.3 3.431 8.731
Chimera Model #9.1
REMARK VINA RESULT: -6.6 0.000 0.000
REMARK 8 active torsions:
REMARK status: ('A' for Active; 'I' for Inactive)
REMARK 1 A between atoms: C_1 and N_9
REMARK 2 A between atoms: C_1 and C_2
REMARK 3 A between atoms: C_2 and N_3
REMARK 4 A between atoms: C_10 and C_11
REMARK 5 A between atoms: C_18 and C_19
REMARK 6 A between atoms: C_21 and N_20
REMARK 7 A between atoms: C_21 and C_22
REMARK 8 A between atoms: C_22 and N_23
Change Compound State
Viable
Deleted
Purged
Hide
Quit
Help

2e

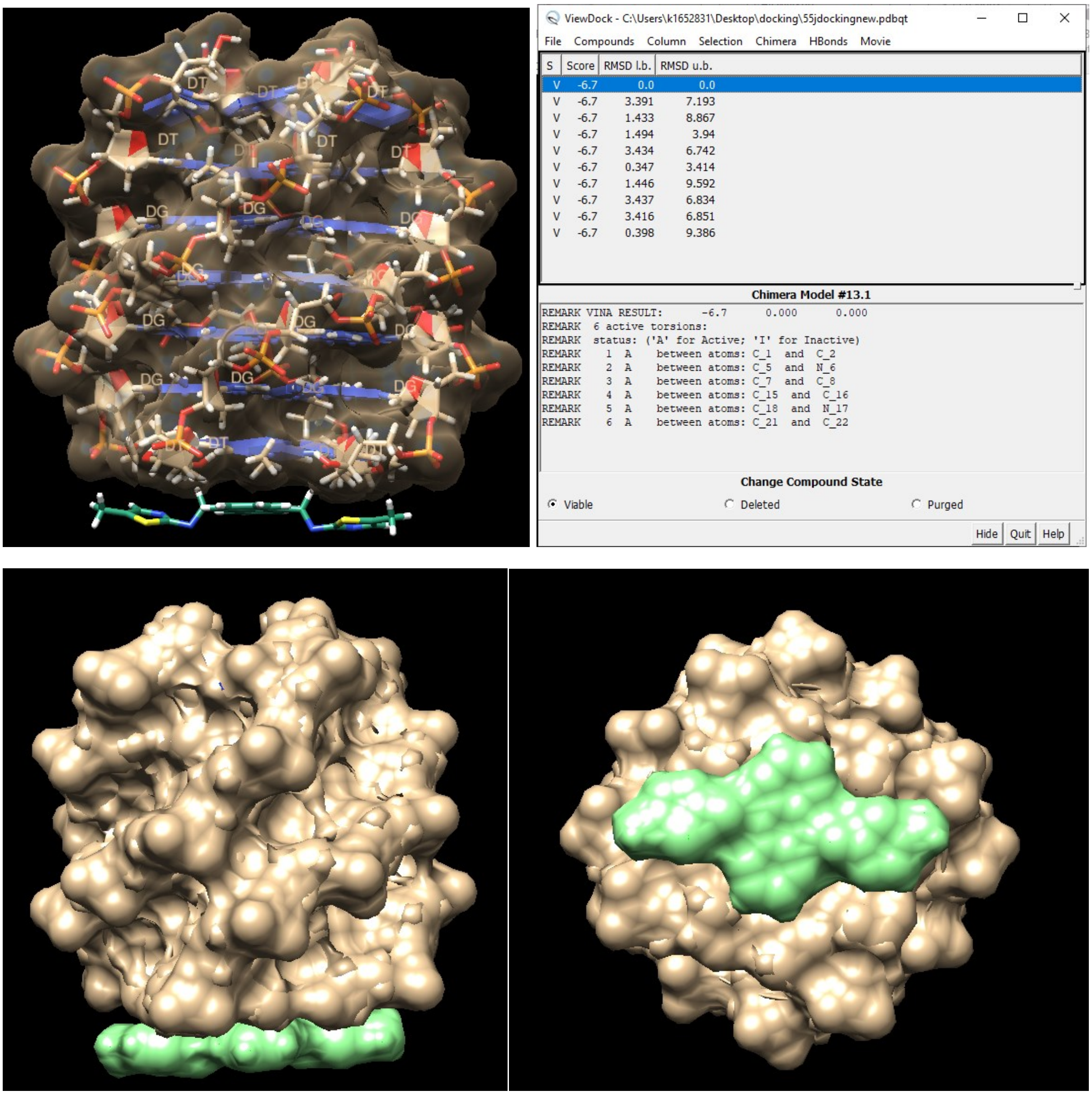
ViewDock - C:\Users\k1652831\Desktop\docking\55jdockingnew.pdbqt
File Compounds Column Selection Chimera HBonds Movie
S Score RMSD l.b. RMSD u.b.
V -6.7 0.0 0.0
V -6.7 3.391 7.193
V -6.7 1.433 8.867
V -6.7 1.494 3.94
V -6.7 3.434 6.742
V -6.7 0.347 3.414
V -6.7 1.446 9.592
V -6.7 3.437 6.834
V -6.7 3.416 6.851
V -6.7 0.398 9.386
Chimera Model #13.1
REMARK VINA RESULT: -6.7 0.000 0.000
REMARK 6 active torsions:
REMARK status: ('A' for Active; 'I' for Inactive)
REMARK 1 A between atoms: C_1 and C_2
REMARK 2 A between atoms: C_5 and N_6
REMARK 3 A between atoms: C_7 and C_8
REMARK 4 A between atoms: C_15 and C_16
REMARK 5 A between atoms: C_18 and N_17
REMARK 6 A between atoms: C_21 and C_22
Change Compound State
Viable
Deleted
Purged
Hide
Quit
Help

Antiparallel d(GGGTTA)4 docking – PDB accession number 139D

2a

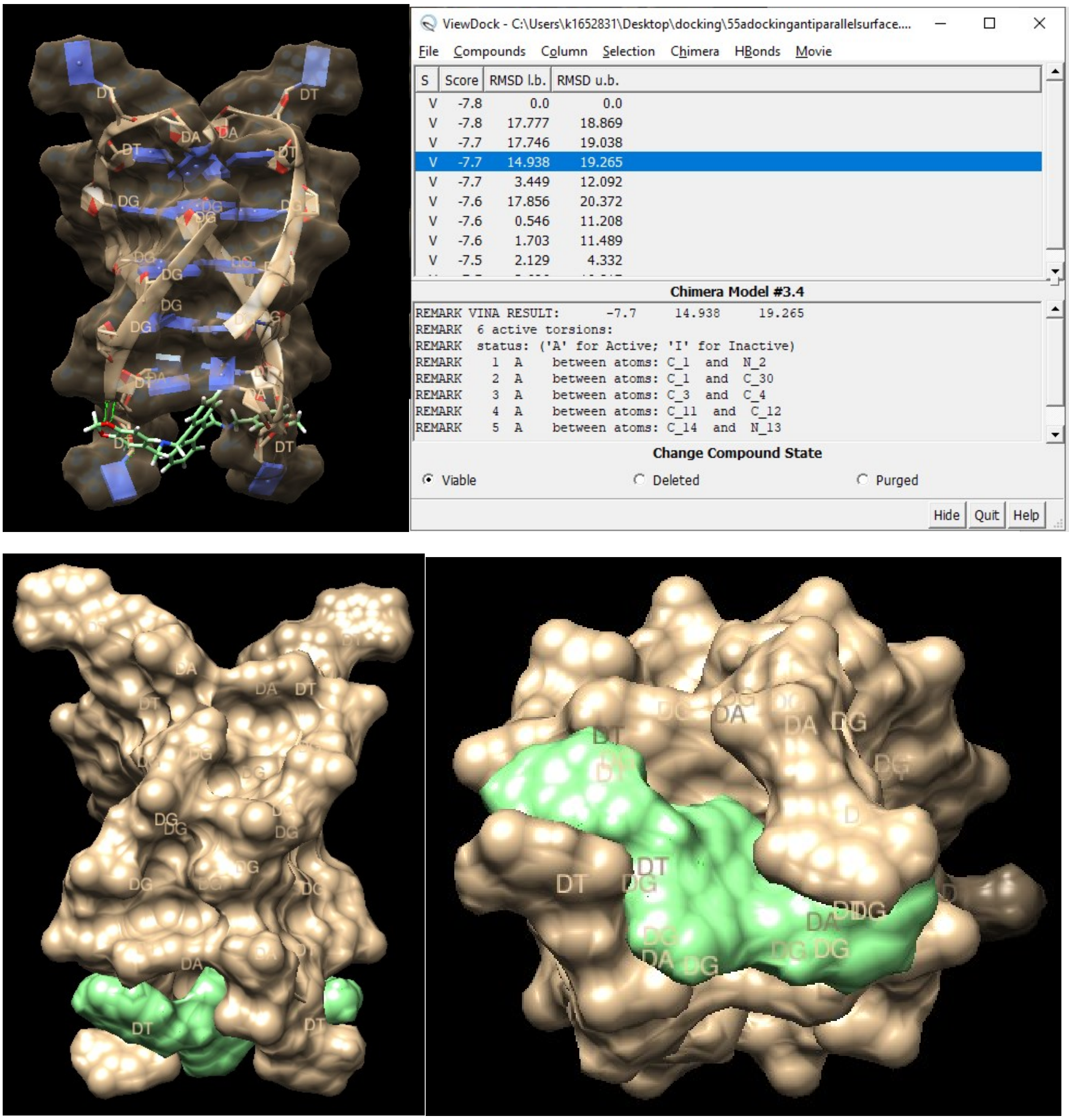

2b

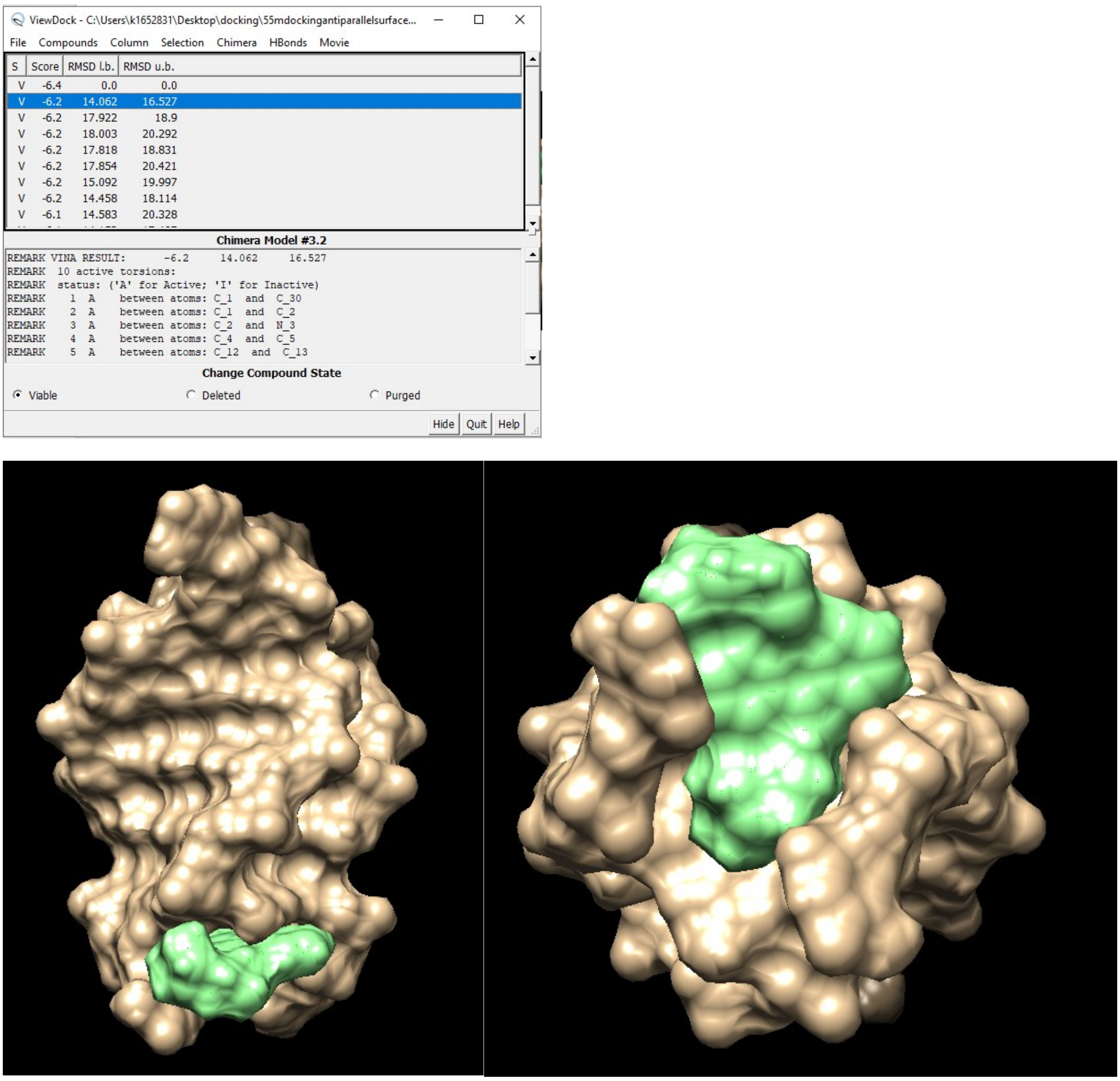
ViewDock - C:\Users\k1652831\Desktop\docking\55mdockingantiparallelsurface...
File Compounds Column Selection Chimera HBonds Movie
S Score RMSD l.b. RMSD u.b.
V -6.4 0.0 0.0
V -6.2 14.062 16.527
V -6.2 17.922 18.9
V -6.2 18.003 20.292
V -6.2 17.818 18.831
V -6.2 17.854 20.421
V -6.2 15.092 19.997
V -6.2 14.458 18.114
V -6.1 14.583 20.328
Chimera Model #3.2
REMARK VINA RESULT: -6.2 14.062 16.527
REMARK 10 active torsions:
REMARK status: ('A' for Active; 'I' for Inactive)
REMARK 1 A between atoms: C_1 and C_30
REMARK 2 A between atoms: C_1 and C_2
REMARK 3 A between atoms: C_2 and N_3
REMARK 4 A between atoms: C_4 and C_5
REMARK 5 A between atoms: C_12 and C_13
Change Compound State
Viable
Deleted
Purged
Hide
Quit
Help

2c

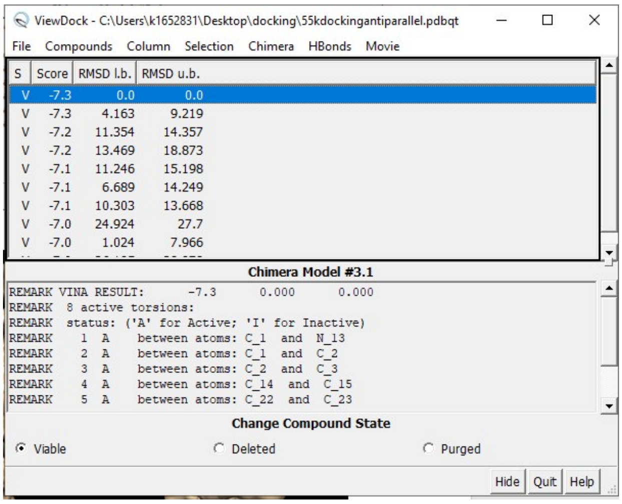
ViewDock - C:\Users\k1652831\Desktop\docking\55kdockingantiparallel.pdbqt
File Compounds Column Selection Chimera HBonds Movie
S Score RMSD l.b. RMSD u.b.
V -7.3 0.0 0.0
V -7.3 4.163 9.219
V -7.2 11.354 14.357
V -7.2 13.469 18.873
V -7.1 11.246 15.198
V -7.1 6.689 14.249
V -7.1 10.303 13.668
V -7.0 24.924 27.7
V -7.0 1.024 7.966
Chimera Model #3.1
REMARK VINA RESULT: -7.3 0.000 0.000
REMARK 8 active torsions:
REMARK status: ('A' for Active; 'I' for Inactive)
REMARK 1 A between atoms: C_1 and N_13
REMARK 2 A between atoms: C_1 and C_2
REMARK 3 A between atoms: C_2 and C_3
REMARK 4 A between atoms: C_14 and C_15
REMARK 5 A between atoms: C_22 and C_23
Change Compound State
Viable
Deleted
Purged
Hide
Quit
Help

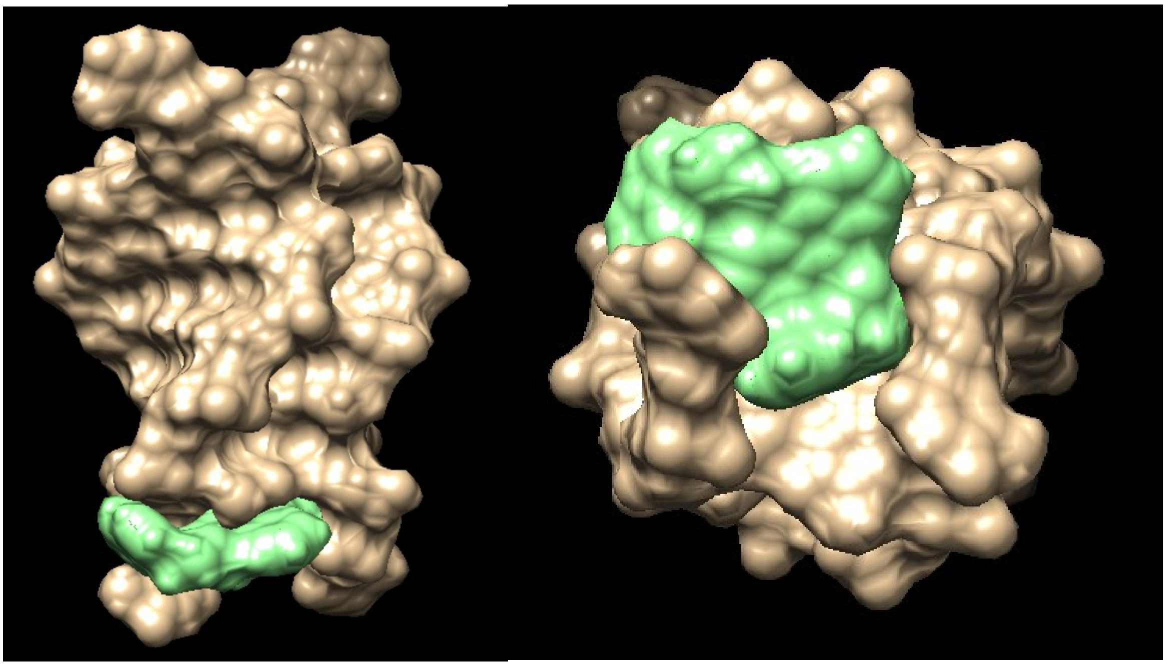

2d

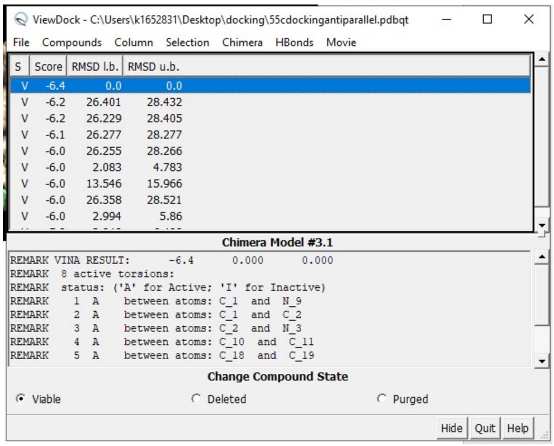

ViewDock - C:\Users\k1652831\Desktop\docking\55cdockingantiparallel.pdbqt
File Compounds Column Selection Chimera HBonds Movie
S Score RMSD l.b. RMSD u.b.
V -6.4 0.0 0.0
V -6.2 26.401 28.432
V -6.2 26.229 28.405
V -6.1 26.277 28.277
V -6.0 26.255 28.266
V -6.0 2.083 4.783
V -6.0 13.546 15.966
V -6.0 26.358 28.521
V -6.0 2.994 5.86
Chimera Model #3.1
REMARK VINA RESULT: -6.4 0.000 0.000
REMARK 8 active torsions:
REMARK status: ('A' for Active; 'I' for Inactive)
REMARK 1 A between atoms: C_1 and N_9
REMARK 2 A between atoms: C_1 and C_2
REMARK 3 A between atoms: C_2 and N_3
REMARK 4 A between atoms: C_10 and C_11
REMARK 5 A between atoms: C_18 and C_19
Change Compound State
Viable
Deleted
Purged
Hide
Quit
Help

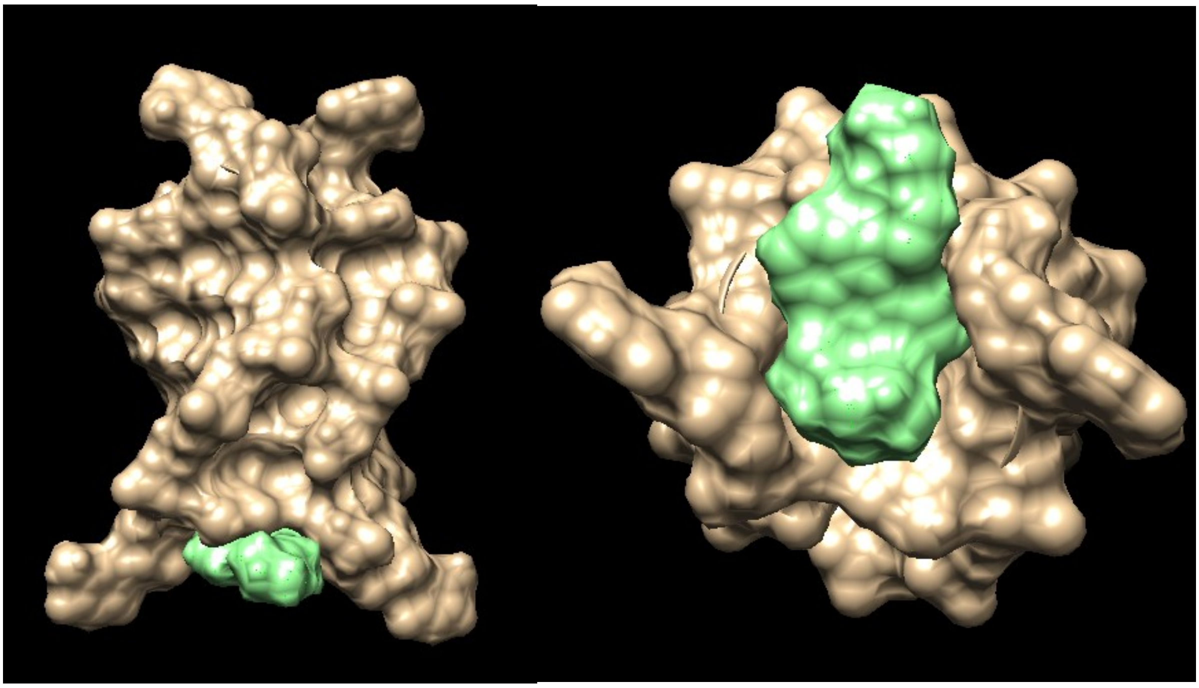

2e

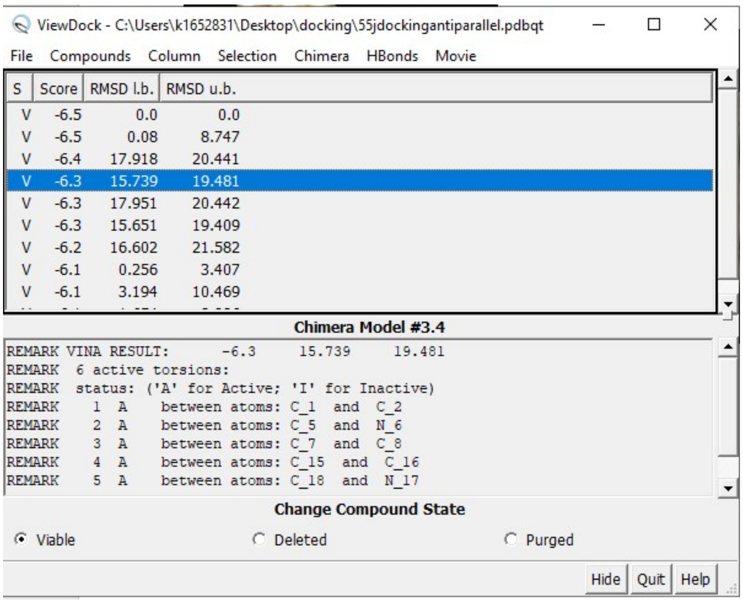
ViewDock - C:\Users\k1652831\Desktop\docking\55jdockingantiparallel.pdbqt
File Compounds Column Selection Chimera HBonds Movie
S Score RMSD l.b. RMSD u.b.
V -6.5 0.0 0.0
V -6.5 0.08 8.747
V -6.4 17.918 20.441
V -6.3 15.739 19.481
V -6.3 17.951 20.442
V -6.3 15.651 19.409
V -6.2 16.602 21.582
V -6.1 0.256 3.407
V -6.1 3.194 10.469
Chimera Model #3.4
REMARK VINA RESULT: -6.3 15.739 19.481
REMARK 6 active torsions:
REMARK status: ('A' for Active; 'I' for Inactive)
REMARK 1 A between atoms: C_1 and C_2
REMARK 2 A between atoms: C_5 and N_6
REMARK 3 A between atoms: C_7 and C_8
REMARK 4 A between atoms: C_15 and C_16
REMARK 5 A between atoms: C_18 and N_17
Change Compound State
Viable
Deleted
Purged
Hide
Quit
Help

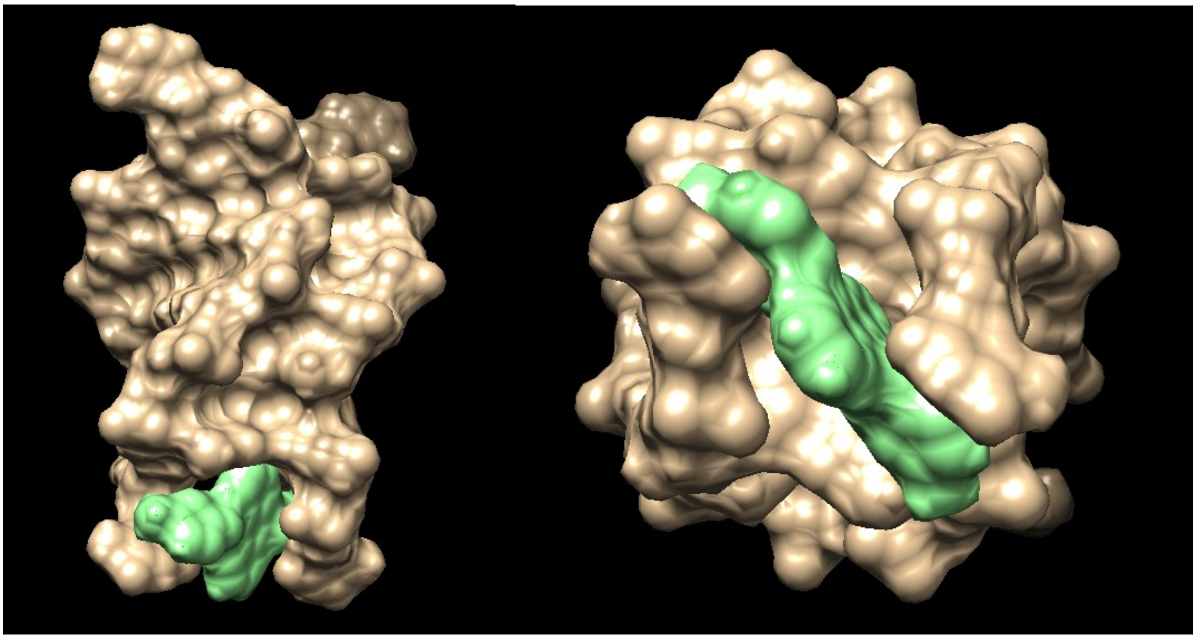

S2. Synthesis supplementary experimental

(E)-N-[2-(1-piperidyl)ethyl]-1-[10-[(E)-2-(1-piperidyl)ethyliminomethyl]-9-anthryl]methanimine

(2d)

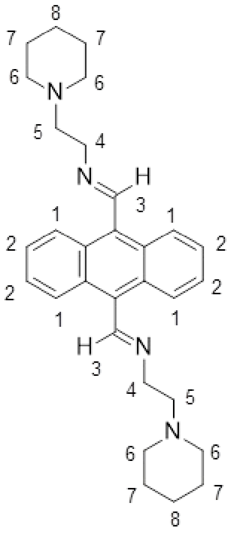

2d

1-(2-Aminoethyl) piperidine (0.35ml, 314mg, 0.0024mol) and 9-10dibromocarbaldehyde (250mg) were stirred in dry DCM (40ml) over molecular sieves. The reaction mixture was refluxed for 12 hrs under N_2 environment. The resulted mixture was monitored *via* TLC which showed the consumption of both starting materials. After TLC analysis, the crude mixture was left to cool down to room temperature. The solid imine product **2d** was filtered and it was dried under vacuum before being recrystallised from methanol to yield a yellow coloured **2d** solid (0.32g, 65% yield) and then it was stored in 0^oC.

^{1}H NMR (400 MHz; d_6-DMSO) δ= 9.3(2H, s, CH-3), 8.5(4H, ddd, *J*=6.90, 3.11, 1.90 Hz, CH-2), 7.52(4H, ddd, *J*=6.98, 3.22, 1.52, CH-1), 4.08(4H, t, *J*=6.96 Hz, CH-4), 2.86(4H, t, *J*=7.70 Hz, CH-5), 2.58(8H, broad, CH-6) 1.67(8H, quintet, *J*=5.46 Hz, CH-7) 1.5 (4H, t, *J*=6.32 Hz, CH-8)

^{13}C-NMR (400 MHz; d_6-DMSO) δ= 24.1, 26.3, 50.9, 54.9, 55, 126.7, 127.4, 130.7, 130.9, 159.9

IR (ATR): v= 2924.30, 2791.03, 2849.60, 1640.34, 1437.78, 754.18

HR MS (ES) m/z = found 455.3179, requires 456.6635 $[M+2H]^+$

MP=159°C

(E)-N-[2-(1H-indol-3-yl)ethyl]-1-[10-[(E)-2-(1H-indol-3-yl)ethyliminomethyl]-9-anthryl]methanimine (2c)

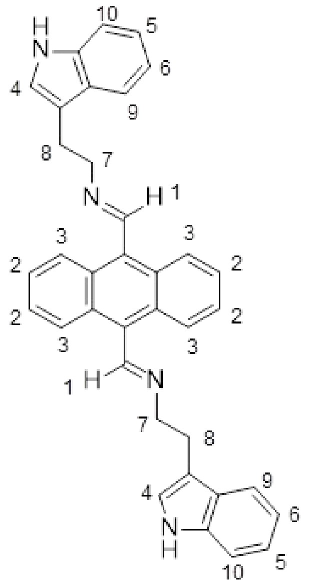

2c

Tryptamine (390mg, 0.00245mol) and 9-10dibromocarbaldehyde (250mg) were stirred in dry DCM (40ml) over molecular sieves. The reaction mixture was refluxed for 12hrs under N_2 environment. The resultant mixture was monitored *via* TLC which showed the consumption of both starting materials. After TLC analysis, the crude mixture was left to cool down to room temperature. The resulting yellow solid **2c** was filtered and it was dried under vacuum before being recrystallised from methanol to give (0.44g, 80%) yield.

^{1}H NMR (400 MHz; d_6-DMSO) δ= 3.68 (4H, t, *J*=6.65 Hz, CH-8) 4.26 (4H, t, *J*=8.66Hz, CH_2-7), 7.01 (2H, t, *J*=8.76Hz, CH-6), 7.10 (2H, t, *J*=8.78Hz, CH-5) 7.23(2H, s, CH-4),7.39 (2H, dddd, *J* = 8.0, 1.2, 0.5, 0.5 Hz*data book values, H-10), 7.424(4H, dd, *J*=6.63, 2.21 Hz, CH-3), 7.695 (2H, d, *J*= 7.74Hz, CH-9), 8.123 (4H, dd, *J*=6.97,2.68, CH-2)

^{13}C-NMR (600 MHz; d_6-DMSO) δ=22.5, 46.9, 111.6, 112, 118.7, 120.2, 121.7, 122.2, 126.7, 127.5, 127.6, 130.7, 130.8, 136.4, 159.9

IR (ATR): v=2843.46, 2922.6, 1652.99, 1621.80, 741.77,755.12

HR MS (ES) m/z = found 519.2541, requires 520.6642 $[M+2H]^+$

MP=238°C

(E)-N-(5-methylthiazol-2-yl)-1-[10-[(E)-(5-methylthiazol-2-yl)iminomethyl]-9-anthryl]methanimine (2e)

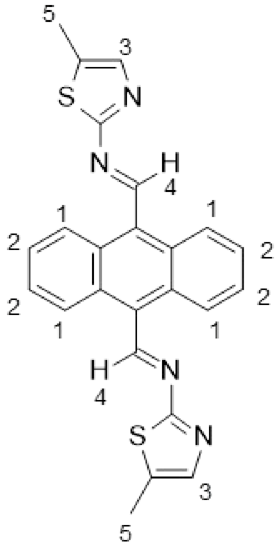

2e

2-Amino-5-methylthiazole (500 mg, 0.0048mol) and 9,10-dibromocarbaldehyde (250mg) were stirred in dry DCM (40ml) over molecular sieves. The reaction mixture was refluxed for 12hrs under N_2 environment. The resultant mixture was monitored *via* TLC which showed the consumption of both starting materials. After TLC analysis, the crude mixture was left to cool down to room temperature. The resulting red solid **2e** was filtered and it was dried under vacuum before being recrystallised from methanol to give (0.34g, 75%) yield.

^{1}H NMR (400 MHz; d_6-DMSO) δ= 1.23 (6H, s, CH_3-5), 7.6 (2H, d, *J*=1.20Hz, CH-3), 7.77 (4H, dd, *J*=6.85,3.24 Hz, CH-1), 8.78 (4H, dd, *J*=6.93,3.19Hz, CH-2), 10.22 (2H, s, CH-4)

^{13}C-NMR (400 MHz; d_6-DMSO) δ=12.495, 124.2714, 128.264, 128.341, 129.366, 129.423, 135.2327, 167.977, 196.115

IR (ATR): v= 2919.38, 1677.56, 1577.16, 1440.95, 751.59

HR MS (ES) m/z = found 427.1048 requires 427.5635 $[M+H]^+$

MP=265°C

(E)-N-(3-morpholinopropyl)-1-[10-[(E)-3-morpholinopropyliminomethyl]-9-anthryl]methanimine (2b)

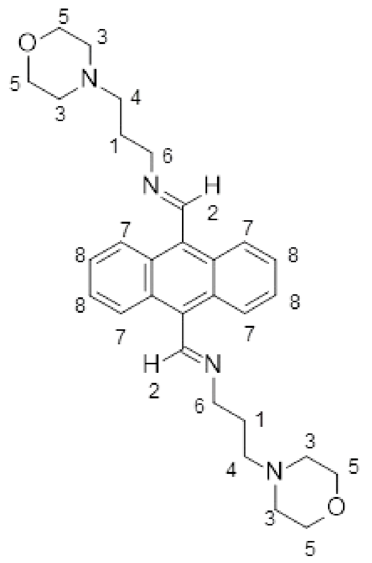

2b

3-Morpholinopropylamine (350 mg, 0.0024mol) and 9,10-dibromocarbaldehyde (250mg) were stirred in dry DCM (40ml) over molecular sieves. The reaction mixture was refluxed for 12hrs under N_2 environment. The resultant mixture was monitored *via* TLC which showed the consumption of both starting materials. After TLC analysis, the crude mixture was left to cool down to room temperature. The resulting off white solid **2b** was filtered and it was dried under vacuum before being recrystallised from methanol to give (0.31g, 60%) yield.

^{1}H NMR (600 MHz; d_6-DMSO) δ= 1.9 (4H, m(5), *J*=7.2Hz, CH-1), 2.29 (2H, s, *data booked values CH-2), 2.39 (4H, q, *J*=1.84Hz, CH-4), 2.52 (4H, t, *J*=1.90Hz, CH-3), 3.49 (4H, t, *J*=4.75Hz, CH-6), 3.62 (8H, t, *J*=4.69Hz, CH-5), 7.60 (4H, dd, *J*=6.69, 3.16 Hz, CH-7), 8.5(4H, dd, *J*=7.24,3.24 Hz, CH-8)

^{13}C-NMR (600 MHz; d_6-DMSO) δ= 23.1, 46.9, 50.9, 54.1, 66.3, 126.7, 126.9, 130.7, 130.8, 159.9

IR (ATR): v= 2807.34, 2854.18, 1630.97, 1238.90, 1116.07.

HR MS (ES) m/z = found 487.3073, requires 488.6623 $[M+2H]^+$

MP=161°C

(E)-N-(1,3-benzodioxol-5-ylmethyl)-1-[10-[(E)-1,3-benzodioxol-5-ylmethyliminomethyl]-9-anthryl]methanimine (2a)

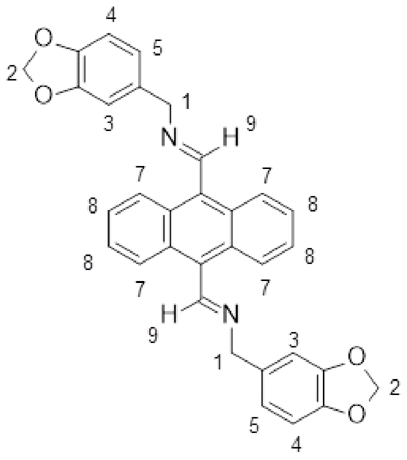

2a

Piperonylamine (370 mg, 0.00245mol) and 9,-10-dibromocarbaldehyde (250mg, moles) were stirred in dry DCM (40ml) over molecular sieves. The reaction mixture was refluxed for 12hrs under N_2 environment. The resultant mixture was monitored via TLC which showed the consumption of both starting materials. After TLC analysis, the crude mixture was left to cool down to room temperature. The resulting yellow solid **2a** was filtered and it was dried under vacuum before being recrystallised from methanol to give (0.42g, 78%) yield.

^{1}H NMR (600 MHz; d_6-DMSO) δ= 5.03 (4H, s, CH-1), 6.02 (4H, s, CH-2), 6.9 (2H, dd, *J*=7.98, 0.42 Hz, CH-4), 6.98 (2H, dd, *J*=7.76, 1.66 Hz, CH-5), 7.08 (2H, dd, *J*=1.42, 0.28 Hz, CH-3), 7.6(4H, dd, *J*=7.04, 3.31 Hz, CH-7), 8.54(4H, dd, *J*=7.01, 3.29 Hz, CH-8), 9.62(2H, t, *J*=1.30Hz, CH-9)

^{13}C-NMR (600 MHz; d_6-DMSO) δ= 65, 101.5, 108.1, 108.9, 125.42, 126.7, 127.6, 130.7, 130.8, 137.5, 147.7, 147.9, 159.9

IR (ATR): v= 2935.03, 1618.90, 1502.29,1488.79,765.11

HR MS (ES) m/z = found 501.1815, requires 501.5509 $[M+H]^+$

MP=236°C
